# HIV gp120 Binds to Mannose Receptor on Vaginal Epithelial Cells and Induces Production of Matrix Metalloproteinases

**DOI:** 10.1371/journal.pone.0028014

**Published:** 2011-11-22

**Authors:** Sashaina E. Fanibunda, Deepak N. Modi, Jyotsna S. Gokral, Atmaram H. Bandivdekar

**Affiliations:** 1 Department of Biochemistry, National Institute for Research in Reproductive Health, Indian Council of Medical Research, Mumbai, Maharashtra, India; 2 Molecular and Cellular Biology Laboratory, National Institute for Research in Reproductive Health, Indian Council of Medical Research, Mumbai, Maharashtra, India; 3 Division of Clinical Research, National Institute for Research in Reproductive Health, Indian Council of Medical Research, Mumbai, Maharashtra, India; German Primate Center, Germany

## Abstract

**Background:**

During sexual transmission of HIV in women, the virus breaches the multi-layered CD4 negative stratified squamous epithelial barrier of the vagina, to infect the sub-epithelial CD4 positive immune cells. However the mechanisms by which HIV gains entry into the sub-epithelial zone is hitherto unknown. We have previously reported human mannose receptor (hMR) as a CD4 independent receptor playing a role in HIV transmission on human spermatozoa. The current study was undertaken to investigate the expression of hMR in vaginal epithelial cells, its HIV gp120 binding potential, affinity constants and the induction of matrix metalloproteinases (MMPs) downstream of HIV gp120 binding to hMR.

**Principal Findings:**

Human vaginal epithelial cells and the immortalized vaginal epithelial cell line Vk2/E6E7 were used in this study. hMR mRNA and protein were expressed in vaginal epithelial cells and cell line, with a molecular weight of 155 kDa. HIV gp120 bound to vaginal proteins with high affinity, (Kd = 1.2±0.2 nM for vaginal cells, 1.4±0.2 nM for cell line) and the hMR antagonist mannan dose dependently inhibited this binding. Both HIV gp120 binding and hMR exhibited identical patterns of localization in the epithelial cells by immunofluorescence. HIV gp120 bound to immunopurified hMR and affinity constants were 2.9±0.4 nM and 3.2±0.6 nM for vaginal cells and Vk2/E6E7 cell line respectively. HIV gp120 induced an increase in MMP-9 mRNA expression and activity by zymography, which could be inhibited by an anti-hMR antibody.

**Conclusion:**

hMR expressed by vaginal epithelial cells has high affinity for HIV gp120 and this binding induces production of MMPs. We propose that the induction of MMPs in response to HIV gp120 may lead to degradation of tight junction proteins and the extracellular matrix proteins in the vaginal epithelium and basement membrane, leading to weakening of the epithelial barrier; thereby facilitating transport of HIV across the vaginal epithelium.

## Introduction

The global HIV-1 epidemic is fuelled through sexual transmission with women accounting for more than half of the 33 million individuals infected with the virus [1]. The lower female reproductive tract, is the initial site of contact with semen containing cell free and cell-associated virus that have been documented to transmit infection *in vivo* (in macaque studies) [2]–[5]. Although HIV can infect the vaginal, ectocervical and endocervical mucosa, the relative contribution of each site to the establishment of infection is not known. The columnar epithelium lining the transformation zone of the endocervix is single layered and thought to be vulnerable to infection [2]; while the stratified squamous epithelium lining the ectocervix/vagina is multi-layered and is believed to offer protection against pathogens when intact [6]–[8]. However, the greater surface area of the vagina/ectocervical wall provides more potential access sites for HIV entry, particularly when breaches occur in the epithelial-cell layer. This is of importance in light of the observation that HIV transmission can occur solely through the vagina in the absence of the endocervix and the uterus [9], [10]. Moreover, anatomically in the vagina, the HIV infected cells include the intraepithelial langerhans cells, T cells [11], as well as dendritic cells, macrophages and T cells that are found in the sub-epithelium or lamina propria below the stratified squamous epithelial layer [12]. While it is plausible that the langerhans cells may extend their projections to the surface, to directly sample HIV from the lumen; HIV must also breach though the robust multilayered vaginal epithelial barrier (25–40 layer thick) to infect the deeply embedded CD4+ immune cells [2], [12]. Thus, any aberrations in the integrity of the epithelial barrier would increase susceptibility to HIV infection. However the mechanisms by which HIV gains entry into the sub-epithelial zone is hitherto unknown.

While the epithelial cells are refractory to HIV entry [11], [13]–[15]; the intact epithelial barrier is impermeable to particles above 30 nm diameter, with the HIV virus estimated to have a diameter of ∼80–100 nm [8]. However, studies have demonstrated that HIV penetrates interstitially between epithelial cells of the stratified squamous epithelium as early as 2 hr [3], [6], [14]. These observations rule out the possibility of HIV being transmitted via the classical replication based mechanisms. Although transcytosis of HIV through the epithelial cells has been reported, the extent is estimated to be very low [16]. Therefore, there must exist alternative mechanisms by which HIV must be able to breach the vaginal epithelial layer.

We and others have previously reported hMR as a CD4 independent receptor playing a role in HIV transmission in different cell types including spermatozoa [17]–[19]. In human astrocytes, HIV binds to hMR and activates MMPs, which in turn degrade the extracellular matrix proteins [20]. In case of primary genital epithelial cells, HIV has also been reported to decrease the expression of tight junction proteins and increase the leakiness of the epithelial layer towards HIV [21], [22]. This led us to hypothesize that hMR may exist on vaginal epithelial cells, which might bind to HIV gp120 leading to production of MMPs, facilitating the degradation of junctional proteins and/or the extracellular matrix in general, thereby inducing a disruption of the epithelial layer organization.

To the best of our knowledge, it is unknown whether human vaginal epithelial cells express hMR that may bind HIV gp120 and induce MMP production. In the present study, we aimed to investigate this hypothesis by studying the presence of hMR, its HIV gp120 binding potential and its affinity constants, and finally the ability of HIV gp120 to induce MMP production via hMR.

## Methods

### Human vaginal epithelial cell specimens

#### Ethics Statement

Vaginal epithelial cells from the lateral vaginal wall, were obtained by swab method from women attending the obstetrics and gynecology clinics at NIRRH after written informed consent, and this study and protocol were specifically approved by the ‘National Institute for Research in Reproductive Health Institutional Ethics Committee for Human Subjects’. Women were in the age group of 21–35years, and had regular menstrual cycles (28–35 days). Purity of the vaginal epithelial cells was confirmed by staining for intracellular cytokeratins. Viability was assessed using trypan blue, and samples with greater than 80% viability were included in the study. To test for possible contamination of other cells in the vaginal preparations, RT-PCR for markers specific for lymphocytes (CD3), dendritic cells (CD11c) and macrophages (CD14) was performed using RNA derived from pools of vaginal epithelial cells as template.

### Culture of human vaginal epithelial cell line - Vk2/E6E7

The cell line was obtained from American Type Culture Collection, originally prepared by R. Fichorova (Fearing Research Laboratory, Boston, MA). Cells were grown as described previously [23] in keratinocyte serum free medium (K-SFM) supplemented with 0.1 ng/ml EGF, 50 ug/ml bovine pituitary extract (Invitrogen, Carlsbad, CA) and 0.4 mM CaCl_2_ (Sigma, St. Louis, MO), at 37°C and 5% CO_2_. Cells were split 1∶3 at 60% confluence. The Vk2/E6E7 cells express markers of terminal differentiation (involucrine and cytokeratin 13) just as fully differentiated vaginal cells on the surface of vaginal tissues [23]. This cell line represents a valid model that has been used by many others to study HIV - epithelial interaction events [24]–[28].

### HIV gp120 binding to vaginal proteins and competitive inhibition by mannan

As a source of human vaginal proteins, human vagina protein medley comprising SDS solubilized proteins from normal human vagina was purchased from Clontech (CA, USA). Vk2/E6E7 cells were lysed in protein extraction buffer M-Per reagent (Pierce, Rockford, USA). The binding of gp120 to vaginal proteins was tested as described earlier [19] with minor modifications. Briefly, wells of an ELISA plate were coated overnight at 4°C, with 20 ug of proteins in 100 ul of a 0.1 M carbonate buffer, pH 9.2. Wells were washed in TBS (Tris Buffered Saline) and blocked in 0.3% BSA prepared in TBS containing 0.1% Tween for 2 hr at room temperature. Increasing concentrations of HIV gp120-IIIB HRP conjugated (Immunodiagnostics, Woburn, Ma USA) (0.2 nM–10 nM) were incubated overnight, followed by six TBS washes containing 0.1% Tween-20. Binding was determined using the substrate TMB/H_2_O_2_ and absorbance was read at 450 nm. Wells coated with BSA (bovine serum albumin) or no vaginal proteins served as the negative control. The hMR antagonist mannan (1000 ng/ml to 50 mg/ml) (Sigma) or sucrose (unrelated carbohydrate) was co-incubated with labeled HIV gp120 (100 ng/ml), reacted with vaginal proteins and detected as above. Further, the binding of HIV gp120 (100 ng/ml), was also studied in the presence of increasing doses of the hMR blocking antibody (clone 19.6 BD Pharmingen, San Diego, CA, USA) or an isotype control IgG, and the binding was quantified as described above.

### hMR expression by RT-PCR and Western blotting

RNA was extracted from vaginal epithelial cells (n = 22, pooled in groups 3–5samples) or the Vk2/E6E7 cell line using the Qiagen RNeasy mini kit, (Qiagen, CA, USA). DNase-treated RNA samples were subjected to RT-PCR. RNA was reverse transcribed to cDNA and amplified with the SuperScript One-Step RT-PCR kit (Invitrogen). Briefly, the reaction mixture of 25 µl contained 0.4 µM each of forward and reverse primers, 200 µM dNTP mix and 1 U SuperScript III RT/Platinum Taq enzyme mix. The reverse transcription was carried out at 55°C for 30 min, followed by denaturation at 94°C for 2 min. This was followed by 35 cycles of amplification, each cycle comprising denaturation at 94°C for 15 s, annealing at 58°C for 30 s and extension at 68°C for 30 s. cDNA was PCR amplified using hMR specific primers– FP: 5′ TAC-ACA-AAC-TGG-GGG-AAA-GG 3′ and RP 5′ TGT-TTG-AAT-CGT-TGC-TGG-AG 3′ as described earlier [17]. The amplification conditions included denaturation at 94°C for 15 s, annealing at 58°C for 30 s, extension at 68°C for 30 s, with a final extension at 68°C for 5 min. The PCR products were sequenced with an automated fluorescence-based sequencer at the core sequencing facility at the institute. Detection of the size of vaginal hMR protein was performed by SDS-PAGE and western blotting of human vaginal protein lysates and Vk2/E6E7 protein lysates. Blots were probed with goat polyclonal anti-hMR serum (1∶1000) (kind gift from Dr. Stahl) as described earlier [17]. Blots probed with normal goat serum served as a negative control.

### HIV gp120 binding and hMR staining by direct fluorescence

Smears of vaginal epithelial cells were processed for HIV gp120-IIIB FITC (Immunodiagnostics) binding (n = 30) and hMR staining (n = 30) as described previously [17]. The cells of the Vk2/E6E7 cell line were grown in K-SFM on glass coverslips and processed similarly. Images were obtained using the Zeiss LSM-510 Meta confocal laser scanning microscope.

### HIV gp120 binding to vaginal hMR

A capture ELISA was developed in the laboratory to study the binding of HIV gp120 to hMR. Briefly, goat anti-hMR polyclonal antibody (1∶1000) in 0.1 M carbonate buffer pH 9.2, was immobilized in wells of an ELISA plate, overnight at 4°C. The wells were washed twice with TBS containing 0.1% Tween (wash buffer), and non-specific binding was blocked using 1% BSA. Human vaginal proteins or Vk2/E6E7 proteins (20 ug) were added to the wells and incubated at 37°C for 1.5 hr, followed by three washes. The ligand HIV gp120 HRP conjugated (100 ng/ml) was added in the presence or absence of the hMR blocking antibody (clone 19.6 BD Pharmingen, San Diego, CA, USA) and detected as described above. Wells coated with normal goat serum and wells containing no vaginal proteins served as controls in the experiment.

### Affinity constants of hMR for HIV gp120

The affinity constants of HIV gp120 to hMR were determined by competitive ELISA optimized in the laboratory. Briefly, 3.3 nM of HIV gp120-IIIB (Immunodiagnostics) in 0.1 M carbonate buffer pH 9.2, was immobilized in wells of an ELISA plate, overnight at 4°C. The wells were washed twice with TBS and vaginal proteins or Vk2/E6E7 proteins (20 ug/well) were incubated at 37°C for 1.5 hr. To determine the affinity constants, the vaginal or the cell line proteins were mixed with increasing amounts of gp120 (0.083 nM to 40 nM) and then added to the wells. The wells were then washed to remove unbound proteins, and incubated with anti-hMR polyclonal antibody (1∶1000) for 2 hr at 37°C to detect the amounts of hMR bound to the immobilized gp120 in the wells. The wells were washed again to remove the excess antibody and then detected using the rabbit anti-goat HRP conjugated secondary antibody (1∶5000) (Dako, Glostrup, Denmark). HRP assay was performed as described above and the OD was read at 450 nm. The negative control comprised of wells that had BSA in place of immobilized gp120; wells which did not contain vaginal proteins served as an additional control.

### Measurement of MMP mRNA levels and activity

For the experiments on MMPs and TIMPs, cells were seeded at density of 1×10∧6 cells/well in six well tissue culture plates (Fisher Scientific, Hampton, NH) or 4×10∧4 cells/well in 96 well plates. Confluent Vk2/E6E7 cells were treated with HIV gp120 (0.083 nM to 83 nM). These concentrations were chosen based on the affinity constants of hMR determined herein and have been reported to be used by others in different cell types [29]–[32]. In some experiments in 96 well plates, along with HIV gp120 (83 nM), cells were coincubated with hMR antibody clone 19.2 or isotype matched IgG. Cell supernatants were collected after 24 hr and assayed for MMP activity by zymography using 1 mg/ml gelatin as a substrate as described [33]. Briefly, samples were electrophoresed through a 10% polyacrylamide gel containing 1 mg/ml gelatin. After electrophoresis, gelatin gels were equilibrated for 30 min at room temperature with zymogram renaturing buffer −2.7% Triton X-100 with gentle agitation. Zymograms were incubated for 12 h at 37°C in zymogram developing buffer −200 mM NaCl, 5 mM CaCl_2_, 0.02% Brij 35, and 50 mM Tris pH 7.5. Gels were stained with 0.5% Coomassie Blue at for 1 hr, destained with methanol∶glacial acetic acid∶water (50∶10∶40) till clear bands were obtained against a blue background. Bands corresponding to MMP-9 and MMP-2 were identified based on their molecular weight and were quantitated by densitometry (Syngene, Cambridge, United Kingdom).

Cell lysates were used to measure MMPs at the mRNA level by real time PCR. RNA was extracted as described above and reverse transcribed to prepare cDNA using the Advantage RT-for-PCR kit (Clontech, CA, USA). Briefly, 1 ug of RNA was heat denatured at 70°C for 2 min, and then reverse transcribed using MMLV reverse transcriptase and 20 pmol random hexamers, 10 mM dNTPs, in a 20 ul reaction volume; at 42°C for 1 hr. Real time PCR was performed, using TaqMan (Applied Biosystems, Foster City, CA) gene expression primer and probe sets on the ABI Prism 7900HT (Applied Biosystems). Briefly, a 20 ul reaction mixture contained 2 ul of cDNA, 1× TaqMan primer probe mix diluted in TaqMan universal PCR master mix and each reaction was set in duplicates. Cycling was carried out at 50°C for 2 min, 95°C for 10 min, and 40 cycles of 95°C for 15 s with an annealing and extension at 60°C for 1 min. A no-template control (H_2_O control) was analyzed for each master mix. All experiments were repeated in triplicates. Levels of 18S rRNA were used for normalization. All probes were FAM labeled except 18S which was VIC labeled. The relative expression ratios were calculated manually using Pfaffl method [34].

### Data analysis and statistical methods

Dissociation constant Kd values were computed using Graph-Pad™ Prism software (San Diego, CA). Data were evaluated using unpaired Students t-test. p<0.05 was accepted as statistically significant.

## Results

### Binding of HIV gp120 to vaginal proteins and effect of mannan on binding

**Figure 1:**
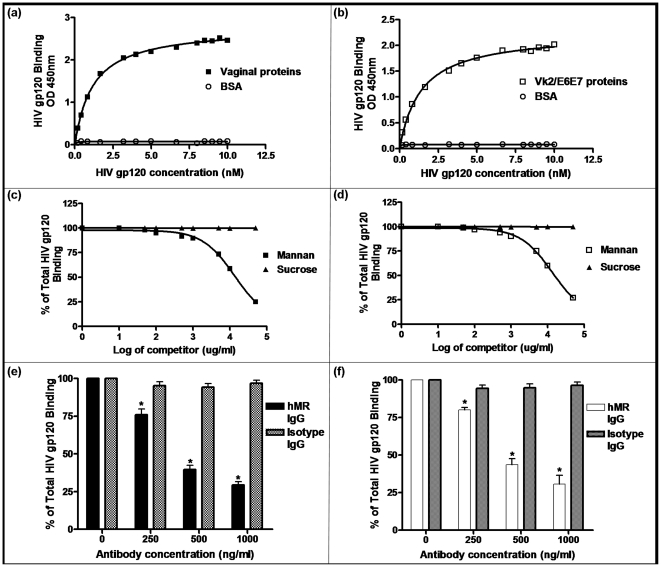
Binding of HIV gp120 to vaginal proteins and its inhibition by mannan or the hMR blocking antibody. Saturation isotherm of HIV gp120 HRP conjugated binding at the indicated concentrations on the X axis to (a) vaginal protein lysates (Kd = 1.2±0.2 nM) and (b) vaginal epithelial cell line Vk2/E6E7 protein lysates (Kd = 1.4±0.2 nM). Results are representative of three experiments. Inhibition of HIV gp120 binding using mannan in (c) vaginal protein lysates or (d) Vk2/E6E7 protein lysates. Mannan or sucrose concentrations in ug/ml are plotted on a logarithimic scale. Experiments were repeated in triplicates. Binding of HIV gp120 to (e) vaginal protein lysates or (f) Vk2/E6E7 protein lysates in the presence of increasing concentrations of hMR blocking antibody or isotype matched IgG antibody. Results are mean ± SE, n = 3. ^*^Statistically significant (p<0.05) when compared with cells treated with HIV gp120 in the absence of antibody.

The binding of HIV gp120 to vaginal proteins, was found to be of high affinity and saturatable (Fig. 1a and b). The affinity of HIV gp120 binding to human vaginal proteins was computed to be 1.2±0.2 nM and 1.4 nM±0.2 nM for the Vk2/E6E7 cell line. BSA at identical or 100× concentrations did not demonstrate gp120 binding.

Mannan (an hMR antagonist), dose dependently inhibited the binding of HIV gp120 to vaginal proteins of either sources, while sucrose (unrelated carbohydrate) had no effect. Mannan at doses ranging from 0.1 mg/ml to 50 mg/ml significantly reduced HIV gp120 binding to human vaginal protein lysates (Fig. 1c) and Vk2/E6E7 lysates (Fig. 1d). At the highest concentration mannan inhibited gp120 binding by almost 75%, indicating that HIV gp120 potentially binds to hMR.

The hMR blocking antibody was found to inhibit the binding of HIV gp120 to vaginal proteins (Fig. 1e), and Vk2/E6E7 proteins (Fig. 1f) by almost 60%. An unrelated isotype matched IgG did not influence the binding, thus demonstrating the specificity.

### Expression of hMR in vaginal cells

**Figure 2:**
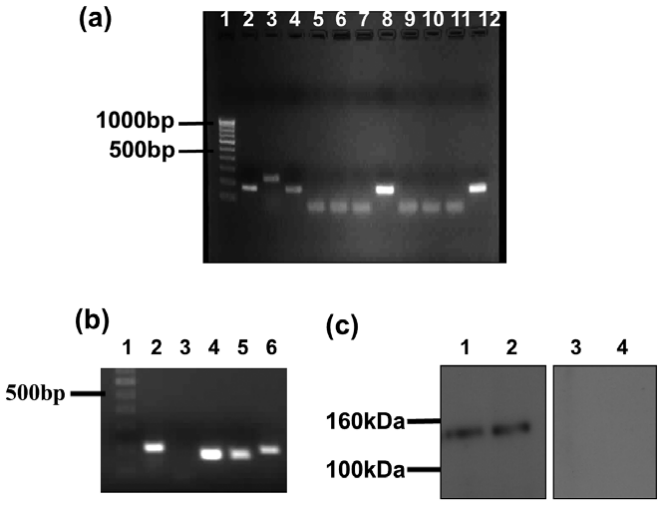
Expression of hMR in vaginal cells. (a) Purity of vaginal epithelial cells: RT-PCR for lymphocyte marker CD3 (lane 2, 5 9), macrophage/monocyte marker CD14 (lanes 3, 6, 10) and dendritic cell marker CD11c (lanes 4, 7 and 11) using RNA isolated form PBMCs (lane 2, 3, 4), epithelial cells (lanes 5, 6, 7) and Vk2/E6E7 (lanes 9, 10, 11). Lanes 8 and 12 - positive control (18S rRNA) from vaginal epithelial cells and Vk2/E6E7 cells respectively. Lane 1 is 100 bp ladder. (b) RT-PCR for hMR mRNA: Lane 1: 100 bp DNA ladder. Lanes 2 and 6: Amplification of hMR in RNA derived from vaginal epithelial cells and Vk2/E6E7 cells respectively using hMR specific primers (201 bp product). Lanes 4 and 5: positive control (18S rRNA) from vaginal epithelial cells and Vk2/E6E7 cells respectively. Lane 3: negative control (no template). (c) Western blot for hMR protein: Lane 1: human vaginal protein lysate and lane 2: Vk2/E6E7 protein lysate were probed with goat polyclonal anti-hMR serum (1∶1000) and HRP conjugated donkey anti-goat secondary antibody (1∶5000), and detected by chemiluminescence. Lane 3: human vaginal protein lysate and lane 4: Vk2/E6E7 protein lysate were probed with normal goat serum (negative controls) and detected as in lanes 1 and 2.

The vaginal epithelial cells were collected by gentle scraping of the lateral vaginal walls and were always observed under a microscope for possibility of other contaminating cells. Most of our preparations were free of any visible contamination; samples where any contaminating cells were visible, were excluded from the study. The purity was also evident by RT-PCR where RNA isolated from 5 independent preparations (and also other pools) did not show presence of transcripts for CD3, CD14 and CD11c which are markers for lymphocytes, macrophages and dendritic cells respectively (Fig. 2a). Bands of expected sizes were however detected in RNA isolated from PBMCs (peripheral blood mononuclear cells) that was used as a positive control. 18S rRNA was detected in all the samples.

RT-PCR using *hMR* specific primers resulted in the amplification of a product of expected size (201 bp) and sequence that matched with 99% identity to macrophage *hMR*, when RNA from vaginal epithelial cells or Vk2/E6E7 cells was used as a template (Fig. 2b). In western blots of human vaginal proteins or Vk2/E6E7 cell lysates, probed with goat anti-hMR polyclonal serum, a single band of ∼155 kDa was detected. No bands were observed when blots were probed with normal goat serum (Fig. 2c).

### Localization of HIV gp120 Binding and hMR expression in vaginal cells

**Figure 3:**
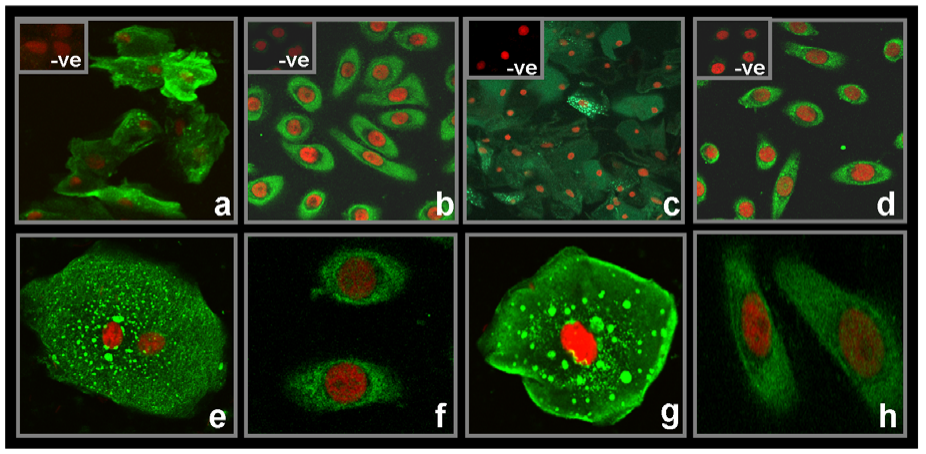
Localization of HIV gp120 binding and hMR in vaginal epithelial cells. Vaginal epithelial cells (a and e) and Vk2/E6E7 cells (b and f) incubated with FITC labeled HIV gp120 (green fluorescence) and counter stained with propidium iodide (red fluorescence). Vaginal epithelial cells (c and g) and Vk2/E6E7 cells (d and h) probed with hMR antibody (green fluorescence) and counter stained with propidium iodide (red fluorescence). *Inset* on (a) and (b) represents vaginal epithelial cells and Vk2/E6E7 cells respectively, incubated with excess unlabelled gp120. *Inset* on (c) and (d) represents negative control (-ve), for vaginal epithelial cells and Vk2/E6E7 cells respectively labeled with IgG antibody FITC labeled. Images (a and c) are at 400× magnification, (b and d) are at 630× magnification. (e–h): Images captured at 1260× magnification and further digitally magnified.

HIV gp120-IIIB FITC binding to human vaginal epithelial cells is depicted in Figure 3a and 3e. The staining in vaginal epithelial cells was found to be heterogeneous and of varying intensities. This binding was specific as the fluorescence staining disappeared when the cells were coincubated with excess of unlabeled gp120 (inset Fig. 3a). In case of Vk2/E6E7 cell line, gp120 bound homogenously to all the cells (Fig. 3b and 3f), which was also displaced by unlabeled protein (inset Fig. 3b).

hMR expression on human vaginal epithelial cells (Fig. 3c and 3g) and the cell line (Fig. 3d and 3h) exhibited identical patterns of staining as observed for HIV gp120 binding. No staining was observed when anti-IgG FITC antibody was used instead of anti-hMR FITC antibody (inset Fig. 3c and 3d).

The heterogenous punctate staining pattern for HIV gp120 binding as well as hMR expression in vaginal epithelial cells was observed across several samples (n = 30 in each case); with a total of ∼95% cells exhibiting staining in each sample. Experiments on the Vk2/E6E7 cell line were repeated in triplicates.

### Binding of HIV gp120 to vaginal hMR

**Figure 4:**
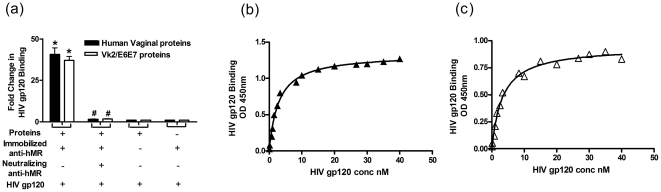
HIV gp120 binding to vaginal hMR and its affinity constants. (a) HIV gp120 binding and its inhibition to immunopurified hMR from human vaginal proteins and Vk2/E6E7 proteins. Results are mean ± SE, n = 3. *Statistically significant (p<0.05) when compared with controls. ^#^Statistically significant (p<0.05) when compared with HIV gp120 treated cells. Saturation Isotherm of HIV gp120 binding to hMR immunopurified from (b) human vaginal proteins and (c) Vk2/E6E7 proteins at concentrations indicated on the X axis. Affinity constants, Kd = 2.9±0.4 nM and Kd = 3.2±0.6 nM for human vaginal proteins and Vk2/E6E7 proteins respectively. Results are a representative of three independent experiments.

Wells coated with anti-hMR polyclonal antibody and incubated with human vaginal proteins or Vk2/E6E7 lysates, exhibited an almost forty fold higher binding of HIV gp120 as compared to negative controls (normal goat serum or no vaginal protein controls). This binding of HIV gp120 to immunopurified hMR was specific, as this binding was reduced to levels comparable to negative controls, in the presence of a neutralizing monoclonal anti-hMR antibody (Fig. 4a).

### Affinity constants of hMR for HIV gp120

To determine the affinity constant of hMR for HIV gp120, a competition ELISA was utilized. In step 1, hMR from vaginal proteins bound to immobilized gp120 in the well and represents ‘Total hMR binding’. Step 2 involved binding of vaginal proteins with increasing amounts of free gp120 followed by its competition with immobilized gp120. hMR not complexed with free gp120 would bind to the immobilized gp120 in the wells. In step 3 the amount of hMR bound to the immobilized gp120 was estimated. A concentration dependent reduction in hMR binding was observed. To obtain the amounts of gp120 complexed to hMR in step 2, the value from step 3 was subtracted from that observed for Total hMR binding (step 1). These values (obtained at each concentration) were used to compute the affinity constant (Fig. 4b and 4c). HIV gp120 binding to hMR was found to be concentration dependent and saturatable. The Kd value (dissociation constant) was computed to be 2.9±0.4 nM (Fig. 4b) from human vaginal proteins and 3.2±0.6 nM (Fig. 4c) from Vk2/E6E7 cell line.

### HIV gp120 induces MMP-9 production

**Figure 5:**
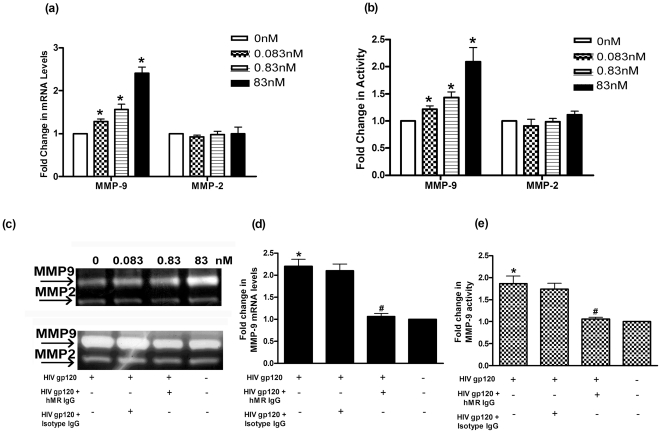
Effect of HIV gp120 on MMP production. (a) Effect of increasing concentrations of HIV gp120 on mRNA expression of MMP-9 and MMP-2 in Vk2/E6E7 cells. (b) Effect of increasing concentrations of HIV gp120 on MMP-9 and MMP-2 activity. (c) Representative zymograms of HIV gp120 treated Vk2/E6E7 cells (upper panel); lane 1: untreated cells, lane 2: 0.083 nM of HIV gp120, lane 3: 0.83 nM of HIV gp120, lane 4: 83 nM of HIV gp120. Lower panel represents effect of anti-hMR antibody on activity of MMP-9 and MMP-2; lane 1: Cells treated with HIV gp120 (83 nM), lane 2: HIVgp120 (83 nM) + isotype IgG, lane 3: HIV gp120 (83 nM) + anti-hMR IgG, lane 4: untreated cells. (d) Effect of anti-hMR antibody on expression of HIV gp120 induced MMP-9 mRNA. (e) Effect of anti-hMR antibody on MMP-9 activity. (a) and (d) values are mean ± SE of relative expression normalized to 18S rRNA from 3 independent experiments. (b) and (e) are mean ± SE of MMP activity expressed as fold change over unstimulated cells from 3 independent experiments. *Statistically significant (p<0.05) when compared with controls. ^#^Statistically significant (p<0.05) when compared with HIV gp120 treated cells.

Treatment of Vk2/E6E7 cells with increasing concentrations of HIV gp120, resulted in a dose dependent and significant increase in the mRNA expression of *MMP-9* but not *MMP-2* (Fig. 5a). As compared to untreated controls, the *MMP-9* mRNA levels were two fold higher, in the cells treated with 83 nM of HIV gp120. Culture supernatants were assayed for MMP-9 and MMP-2 activity. A single band of ∼92 kDa and ∼72 kDa corresponding to MMP-9 and MMP-2 respectively were obtained on zymograms (Fig. 5c). As compared to the untreated controls, increasing doses of HIV gp120 significantly increased the activity of MMP-9; the activity of MMP-2 was unchanged (Fig. 5b). At 83 nM concentration of HIV gp120, the activity of MMP-9 doubled as compared to untreated controls.

To test if the increase in MMP-9 by HIV gp120, is mediated via hMR, the Vk2/E6E7 cells were incubated with the neutralizing monoclonal blocking antibody (clone 19.2) along with HIV gp120. The anti-hMR antibody significantly inhibited the HIV gp120 mediated increase in MMP-9 mRNA expression (Fig. 5d) and activity (Fig. 5e). This inhibition was specific because an isotype specific mouse IgG did not affect the HIV gp120 mediated increase in MMP-9 transcription and activity.

## Discussion

The present study demonstrates that human vaginal epithelial cells and the Vk2/E6E7 cell line interact with HIV gp120 via the hMR. Mannose receptors on vaginal epithelial cells were found to exhibit high affinity binding to HIV gp120, demonstrated saturatable binding kinetics and were inhibited by the mannose receptor antagonist mannan. Further, we demonstrate that binding of HIV gp120 to hMR increases MMP-9 transcription and activity in the Vk2/E6E7 cells. To the best of our knowledge, this is the first report demonstrating the presence of hMR on human vaginal epithelial cells, and its ability to bind to HIV gp120 leading to MMP production.

The sexual route of HIV transmission accounts for 70–80% of the HIV epidemic with females being disproportionately affected compared to males [1]; yet HIV transmission across the CD4 negative stratified squamous vaginal epithelium of the lower female reproductive tract remains enigmatic. In women treated with nanoxynol-9 which damages the vaginal epithelium, a higher risk of HIV infection is reported [35]; micro-aberrations of the vaginal epithelium due to physical insults or inflammation due to STI infections is also known to increase the infectivity [2], [6]. In a recent study by Horbul *et al*, herpes simplex virus induced disruption of the surface epithelium allowed direct access of HIV to sub-mucosal CD4+ T cells [36]. Thus, the epithelial barrier integrity appears to be a crucial determinant for HIV entry via the sexual route. Studies have demonstrated that the normal vaginal epithelium has limited permeability; the vaginal epithelial cells are connected by tight junctions, adherens junctions and desmosomes that form an intercellular seal to restrict paracellular diffusion of molecules across the epithelial sheet [37]. These epithelial cells (25–40 layer thick) rest on a basement membrane comprised of extracellular matrix proteins which are secreted by the epithelial cells [38]. Hence the incoming virus must traverse through several layers of impervious vaginal epithelium and also breach the basement membrane, before it can reach and infect the target CD4+ immune cells in the sub-epithelium. The fact that HIV particles have been detected interstitially between cells in several layers of the stratified epithelium [7], [14] and also in the lowermost layer of cells of the vaginal epithelium that rests on the basement membrane [7], [11], suggests that there must exist a mechanism by which the virus, would first affect the epithelial cells, and alter their physiology, making them more permeable to eventually allow viral entry and penetration into the sub-epithelial layers. Towards this end, we hypothesized that there must exist some specific receptors for HIV on the human vaginal epithelial cells, which when activated would weaken the epithelial barrier, permitting viral entry to sub-epithelial cells.

Firstly, we studied HIV gp120 binding to human vaginal cell protein lysates and lysates from Vk2/E6E7 cells. HIV gp120 readily bound to proteins from both sources, and this binding was saturatable and of high affinity with Kd values of 1.2±0.2 nM and 1.4±0.2 nM respectively. These results indicate that there exist some high affinity receptors for gp120 on the vaginal epithelium. Vaginal epithelial cells were found to be negative for the classical CD4, CXCR4 and CCR5 receptors (unpublished data). Indeed non-cannonical HIV receptors like syndecans [16] and gp340 [26] have been reported to be present on human vaginal epithelial cells. Beyond these, hMR is a non-cannonical receptor for HIV gp120 [17]–[19]. To test if hMR could be a putative HIV binding protein in human vaginal cells, we tested the effect of the hMR antagonist mannan on HIV gp120 binding. As reported earlier [17]–[19], mannan inhibited the binding of HIV gp120 to vaginal proteins leading us to speculate that HIV gp120 may bind to hMR. To test this further, the binding of HIV gp120 was also tested in the presence of an hMR antibody that is known to neutralize ligand binding. This antibody also reduced gp120 binding to vaginal proteins significantly suggesting that HIV gp120 does bind to hMR in vaginal cells.

At present, the existence of hMR in human vaginal epithelial cells has not been reported. In the present study, for the first time we demonstrated that both hMR mRNA and protein are expressed by vaginal cells and also in the epithelial cell line. *hMR* transcripts were detected by RT-PCR in RNA isolated from vaginal epithelial cells and also from the cell line. The transcript detected in the squamous cells was not from any contaminating lymphocytes, macrophages or dendritic cells indicating that the squamous epithelial cells indeed transcribe *hMR*. In vaginal squamous epithelial cells, we observed a heterogenous punctate or speckled pattern of hMR staining by immunofluorescence, and this pattern replicated when HIV gp120 was used as a probe. hMR has been found to exist in equilibrium, in both monomeric and dimeric forms [39], [40]. It is possible that in the vaginal epithelial cells too, hMR may exist in a multimeric form and may appear as aggregates. However, such punctate pattern of staining was not observed in cell lines, where both hMR and gp120 binding appeared homogenous. At present it is difficult to explain the reason for such differences in staining pattern; it is possible that *in vivo* hMR may have already experienced its ligands, and may have dimerized, which may not occur in case of the cell lines.

The observation that mannan inhibited HIV gp120 binding to vaginal cells as well as the Vk2/E6E7 cell line, and hMR is expressed by the same, prompted us to test if HIV gp120 binds to hMR and determine its binding kinetics. Immunopurified hMR from vaginal proteins of either source bound to gp120, and this binding could be displaced by a blocking anti-hMR antibody. The HIV gp120 binding to hMR was concentration dependent and saturatable with a dissociation constant in the range of 2.9±0.4 nM for human vaginal proteins and 3.2±0.6 nM in the case of Vk2/E6E7 cell line proteins; demonstrating it to be a high affinity gp120 receptor. It is interesting to note that this affinity is within the range reported for hMR from other cells [41], [42]. However, this dissociation constant is lesser than that observed for HIV gp120 binding to total vaginal proteins observed herein. This is not surprising, as beyond hMR other non-cannonical HIV receptors like syndecans and gp340 are also known to be present in vaginal epithelial cells [16], [26].

At present the concentrations of gp120 in the female reproductive tract are not known. *In vivo*, gp120 exists in both soluble forms and virion bound. Gp120 which is labile due to non-covalent linkage with gp41, sheds spontaneously from the surface of virions [43]–[47] and is also released from virus infected cells [44], [48]; giving rise to soluble forms of gp120. On the surface of the virus, the gp120 is known to exist in the form of trimers, and is also reported as monomers, with the monomeric form being highly accessible [43], [49]–[52]. While the affinity constants of the trimeric gp120 to hMR have not been determined; considering the high affinity of monomeric gp120 to hMR, hMR could concentrate the virus on the mucosal surface and may act as a local reservoir. Given the fact that each virion would carry around 24 copies of gp120 [53], [54], accumulation of virus particles above a threshold would possibly activate a range mechanisms pertaining to epithelial cell damage and transcytosis to gain entry in to sub-epithelial layers. It is also possible that the affinity of hMR to trimeric gp120 may be higher than the monomeric form (used herein) which may further potentiate accumulation of the virus in the tract.

*In vivo*, mannosylated proteins and other ligands for hMR are known to trigger a plethora of signaling cascades to elicit biological effects [55]. In astrocytes, it has been demonstrated that under the influence of HIV, hMR activates PI3-kinase signaling leading to production of MMPs [20]. Indeed, an increase in MMPs has been found in various HIV associated pathologies [56]. HIV signaling would largely depend on exposed accessible gp120 at its surface, which contains both trimeric and monomeric forms with the monomeric form though less in quantity is highly accessible [43], [49]–[51]. To test if the binding of monomeric HIV gp120 to hMR may induce the production of MMPs in vaginal epithelial cells, we challenged the Vk2/E6E7 cells with increasing concentrations of gp120 and tested for MMP-2 and MMP-9 transcription and their activities. A dose dependent increase in the mRNA expression and activity of *MMP-9* but not *MMP-2* in response to HIV gp120, was observed. The levels of *TIMP-1, TIMP-2 and TIMP-3* mRNA in gp120 treated and untreated cells were identical (unpublished data). These results imply that HIV gp120 induces MMP-9 production in vaginal epithelial cells. Beyond hMR, the vaginal epithelial cells have other HIV binding proteins like gp340 and syndecans [16], [26]. To test if the induction of MMP-9 by HIV gp120 in the epithelial cells is via hMR or other HIV binding proteins, we tested the effects of an hMR neutralizing antibody on gp120 mediated MMP-9 production. The anti-hMR antibody completely abrogated the HIV gp120 mediated increase in MMP-9 production and the levels were identical to untreated controls. These results indicate that the effect of HIV gp120 on MMP-9 production is specifically via hMR. It will be of interest to determine if the other gp120 binding proteins like syndecans or gp340 also actuate signaling cascades that culminate into activation of proteases. Furthermore, it would be of interest to compare the effects of monomeric and trimeric gp120 on hMR mediated protease production; and determine if the upregulation in proteases observed herein in our study, is further increased by trimeric gp120. These studies using trimeric gp120 merit further investigation.

In the context of the vaginal epithelial layer, the observation that HIV gp120 induces MMP production is pathologically relevant. MMPs are proteolytic enzymes employing a range of substrates like collagen, fibronectin etc. [56]. MMPs are also known to degrade tight junction components, followed by disruption of the overall tight junction complex leading to increased permeability [57]–[60]. Degradation of adherens junction components by MMPs has also been reported [61], [62]. The increased production of MMP-9 in response to HIV gp120 would lead to possible degradation of tight junction proteins, and the extracellular matrix in the vaginal epithelium and the basement membrane, thereby weakening its integrity and permitting entry of viral particles. Supporting our hypothesis of vaginal epithelial barrier leakiness, Nazli et al. [21] reported a decrease in expression of junctional proteins with increased permeability of genital epithelial cells, following HIV gp120 binding. These results together with our observations that HIV gp120 is capable of inducing MMPs, prompt us to hypothesize that HIV itself facilitates its initial entry, by compromising the integrity of the host tissue, thereby gaining access to the deeply embedded immune cells to obtain a permanent residency and replicative potential in the host. This mechanism would gain further significance in women where the epithelial layer integrity is already compromised due to physical insults or infections; further activation of MMPs and/or loss of junctional proteins by the virus would immediately make the barrier extremely vulnerable to HIV entry. Not surprisingly the incidence of HIV infection is known to be very high in women with different causes of epithelial layer damage [2], [6].

In conclusion, this study has identified hMR to be expressed in vaginal epithelial cells and binds HIV gp120 with high affinity. The presence of an HIV binding receptor in the vaginal epithelium that would facilitate MMP production thereby weakening the vaginal epithelial barrier, presents a potential new mechanism for mucosal transmission of HIV. Further delineation of the downstream events in the hMR pathway would lead to a more detailed understanding of the early events in the mucosal transmission of HIV. This would open up newer avenues in the design of microbicides like hMR antagonists or MMP inhibitors to block HIV in its tracks at the portal of entry.
